# Unraveling the Mystery of Breast Cancer Dormancy: Insights into Genetic, Receptor, and Cellular Interactions Driving Late Recurrence

**DOI:** 10.3389/fimmu.2025.1657383

**Published:** 2025-09-04

**Authors:** Haochen Ma, Bingqiang Zhang, Mengmeng Chen, Zhendi Song, Yi Zhao

**Affiliations:** ^1^ Institute for Translational Medicine, The Affiliated Hospital of Qingdao University, College of Medicine, Qingdao University, Qingdao, China; ^2^ Key Laboratory of Cancer and Immune Cells of Qingdao, Qingdao Restore Biotechnology Co., Ltd., Qingdao, China; ^3^ Department of Breast Surgery, Qingdao Municipal Hospital, Qingdao, China

**Keywords:** breast cancer, dormancy, tumor microenvironment, non-coding RNA, cell adhesion molecule

## Abstract

Late recurrence of breast cancer poses a considerable threat to patient survival, often attributed to breast cancer dormancy. Dormancy, characterized by cancer cells remaining quiescent for extended periods, is influenced by genetic factors and modifications that directly impact cellular phenotype. Alterations in gene expression dynamically shape cellular behavior, often mediated through receptor signaling pathways. Moreover, interactions within the tumor microenvironment play a pivotal role, fostering either cancer cell dormancy or promoting their escape from dormancy. This review endeavors to provide a comprehensive synthesis of recent advancements in understanding breast cancer dormancy across genetic, receptor molecular, and cellular dimensions. By elucidating the intricate mechanisms underlying dormancy, we aim to shed light on potential therapeutic strategies to prevent late recurrences. Furthermore, we anticipate future research directions that may uncover novel insights into this complex phenomenon, ultimately improving patient outcomes and refining clinical management strategies for breast cancer recurrence.

## 1 Introduction

Breast cancer is the most prevalent malignancy among women, with its annual incidence rising even as the overall mortality rate decreases. Women aged 20 to 49 experience a significantly higher incidence of breast cancer (73.2 cases per 100,000 individuals) compared to other age groups, and the breast cancer mortality rate in this cohort is more than twice as high as that of other cancers (1, 2). Statistically, there is a specific racial difference in the incidence of breast cancer. Although early detection and treatment can yield favorable outcomes, late recurrence remains the primary cause of death from breast cancer, particularly recurrence after surgery, which renders the initial treatment ineffective (3, 4). Breast cancer often evades the effects of surgery and postoperative treatment through metastasis and dormancy, which results in the survival of tumor cells within small metastases.

Dormancy can persist for years, or even decades, and reactivation of tumor cells by specific stimuli leads to a return to a state of high proliferation, giving rise to late metastatic lesions. Carcinoma *in situ* release thousands of cancer cell into the bloodstream. Blood tests may be able to identify circulating tumor cells (CTCs) once they have entered the bloodstream. The blood artery wall must first be penetrated by tumor cells in order for them to spread into the bloodstream (invasion) (5). Subsequently, CTCs must withstand the shear stress in the bloodstream and the immune surveillance by circulating immune cells. The typical tumor phenotype is insufficient to support the completion of this process, and therefore, epithelial-mesenchymal transition (EMT) is considered essential. EMT is a multifaceted biological program in which polarized epithelial cells lose their apical–basal polarity and intercellular junctions, acquiring an elongated, fibroblast-like morphology. Within the context of cellular quiescence, EMT can facilitate immune evasion by driving cells into a growth-arrested state while simultaneously activating pro-survival pathways. This dual functionality positions EMT as a central mechanism linking tumor dormancy to metastatic dissemination (6). For example, zinc finger protein 281 (ZFP281) is frequently utilized as a marker of mesenchymal transition in breast cancer. The diverse mechanisms by which epithelial–mesenchymal transition (EMT) contributes to tumor progression are outlined in the following section (7, 8). CTCs are referred to as disseminated tumor cells (DTCs) when they infiltrate other organs (9). DTCs in secondary organs are often found to exhibit cancer stem cell (CSC) characteristics, either via extracellular vesicles or direct contact with niche cells (10). CSCs are considered to be the final stage of cancer dormancy. DTCs acquire stem cell properties and subsequently gain immune evasion and reduced proliferative capacity through direct interactions with niche cells in the microenvironment or through binding to exosomes derived from mesenchymal stem cells.

Late-stage disease recurrence is particularly associated with breast cancer dormancy, especially in hormone receptor-positive (HR+) breast cancers (11). It is understood that dormancy is necessary but not sufficient for late recurrence. Patients with luminal A-type breast cancer, characterized by hormone receptor positivity (HR+), particularly estrogen receptor-positive (ER+) and progesterone receptor-positive (PR+), often display low Ki67 expression. In contrast, luminal B-type breast cancers are also HR+, but they may be either human epidermal growth factor receptor 2-positive (HER2+) or HER2-negative, with elevated Ki67 levels. Clinical data suggest that luminal A breast cancers are more likely to harbor DTCs, which predominantly express creatine kinase (CK). Conversely, DTCs in early-stage breast cancers may exhibit varying levels of HER2 expression (12). A report has shown that the long non-coding RNA (lncRNA) ELEANORS is exclusively expressed in ER+ breast cancers, where it has been implicated in relapse-promoting activity and upregulation of a breast cancer stemness gene, CD44, which helps maintain the tumor stem population and dormancy (13). This evidence suggests that ER+ breast cancers are more prone to metastasis and recurrence (14). Preinvasive carcinoma and late-stage breast cancer recurrence are most likely to occur in the bone, with tumors remaining dormant for 20 years or more. Personal lifestyle factors are closely linked to relapse (15). Metabolic disorders such as obesity and diabetes disrupt glucose and lipid homeostasis, leading to aberrant expression of key metabolic enzymes and hormones. For instance, acyl-coenzyme A synthetase long-chain family member 3 (ACSL3) is markedly upregulated in disseminated tumor cells (DTCs), enabling them to evade chemotherapy and adopt a dormant phenotype (16, 17). Age is believed to facilitate the reawakening of cancer cells from dormancy, as evidenced by the long-term recurrence of breast malignancies. Recent studies have suggested that the aging microenvironment promotes the formation of ER+ DTCs, providing a more plausible explanation for the occurrence of long-term recurrences (18, 19).

## 2 What leads to dormancy?

Late recurrence refers to breast cancer metastasis and recurrence that occur many years (or even decades) after successful treatment. Interestingly, the sites of metastasis often indicate that these lesions had already formed before the primary cancer was completely eradicated, despite the primary tumor being eliminated decades earlier. Over these decades, these metastatic cells exhibit a state of quiescence, a condition referred to as breast cancer dormancy. Quiescent cancer cells frequently possess immunosuppressive and stem cell-like characteristics, enabling them to evade immune detection and persist in a dormant state. Within the tumor microenvironment (TME), tumor cells and stromal cells engage in dynamic communication, which plays a critical role in maintaining tumor growth and metastasis. The substances and cells released from the TME can influence the normal metabolism and function of distant organs, creating pre-metastatic niches (PMNs) in sensitive organs. These PMNs act as “landing pads” to facilitate the colonization and growth of metastatic tumor cells. Furthermore, non-coding RNAs (ncRNAs) have been shown to modulate tumor characteristics, while cell surface molecules can influence gene expression through their respective signaling pathways. These mechanisms are all integral to the phenomenon of breast cancer dormancy. In this study, we aim to explore the cellular and molecular mechanisms underlying breast cancer dormancy, with a particular focus on the genetic, receptor-mediated, and intercellular interaction-driven factors that contribute to late recurrence. By unraveling these mechanisms, we hope to gain deeper insights into the biology of breast cancer dormancy and identify potential therapeutic targets for preventing late recurrence.

### 2.1 The cellular level

Long-term research has demonstrated that cancer exhibits dormancy, which is closely associated with the immune system and the tumor-associated microenvironment. At the cellular level, tumor cells interact with immune cells within the TME, where they suppress anti-tumor T cells and phagocytes to facilitate their own development (20). A conventional approach to treating and controlling tumors is chemotherapy. However, emerging research suggests that paclitaxel chemotherapy promotes the infiltration of M2 macrophages and tumor-acclimated neutrophils into the TME, implying a connection between dormancy and the IL-6/G-CSF and MEK/ERK signaling pathways (21). Functionally, interleukin-6 (IL-6) acts directly on dormant breast cancer cells by promoting their re-entry into the cell cycle. Treatment with docetaxel has been shown to increase Ki-67 positivity in cancer cells in an IL-6–dependent manner, while neutralization of IL-6—particularly when combined with granulocyte colony-stimulating factor (G-CSF)—significantly reduces this proliferation-inducing effect. In contrast, G-CSF primarily contributes to the establishment of an immunosuppressive tumor microenvironment, likely through the mobilization and polarization of myeloid-derived cells, thereby facilitating the outgrowth of reactivated tumor cells despite concurrent chemotherapy. In contrast to the TME, a PMN lacks tumor cells but contains tumor-supporting cells (22). Before metastasis, tumor cells *in situ* can regulate the systemic microenvironment and metabolism through mechanisms such as exosomes and miRNAs, creating an environment conducive to tumor colonization and dormancy in more sensitive organs (23, 24). For instance, breast cancer cells secrete miRNA-122 via exosomes to regulate glucose metabolism in PMNs, thereby facilitating breast cancer colonization and even the formation of secondary tumors (25). Specifically, tumor cell–derived miR-122 inhibits the growth of primary tumors but promotes metastasis by suppressing glucose uptake and reducing pyruvate kinase activity, thereby impairing cellular metabolism. Tumor cells secrete large quantities of miR-122–enriched exosomes and targeting miR-122 with antagonists may represent a promising strategy to inhibit metastatic progression. PMNs can also be influenced by environmental and personal factors. For example, nicotine in smoke recruits N2 neutrophils to the lungs of individuals who smoke or are exposed to secondhand smoke over extended periods. These N2 neutrophils activate STAT3-activated lipocalin 2 (LCN2), which facilitates the formation of the tumor microenvironment and supports tumor colonization during metastasis (26). Additionally, triple-negative breast cancer (TNBC) cells exert similar effects by releasing the pluripotent factor LIN28B, which recruits and polarizes N2 neutrophils to promote tumor growth and metastasis (26). Tumor mass dormancy is frequent in breast cancers (27). Evidence has shown that the EMT is critical for early tumor transformation, while the mesenchymal-epithelial transition (MET) governs cell colonization (14, 28, 29). The role of WNT signaling in regulating EMT and advancing cancer dormancy has been extensively studied. During dormancy, EMT and MET serve as a strong foundation for tumor persistence and recurrence (30).

### 2.2 The molecular level

Gene expression plays a critical role in regulating the cell cycle and quiescence processes at the molecular level. Non-coding RNAs, including long non-coding RNAs (lncRNAs) and microRNAs (miRNAs), are key regulators of gene expression. miRNAs can be derived from cellular sources or be transferred via exosomes secreted by neighboring cells. The dormant state is often characterized by specific cell adhesion molecules, which serve as indicators of tumor dormancy. Notably, resting breast cancer cells exhibit reciprocal expression patterns of N-cadherin and E-cadherin, where N-cadherin expression is upregulated while E-cadherin expression is downregulated. This shift in cadherin expression is associated with enhanced invasiveness and metastatic potential. In addition, the leukemia inhibitory factor receptor (LIFR) participates in signaling pathways that promote dormancy and transiently halt tumor progression. During tumor dormancy, the WNT signaling pathway, which is tightly linked to cell cycle regulation, often exhibits aberrant activation or inactivation. Despite significant advances in understanding breast cancer dormancy, the molecular mechanisms underlying this phenomenon remain poorly defined. This review focuses on the molecular mechanisms, particularly those involving genetics, epigenetics, cell adhesion molecules, and immune cell interactions, that govern breast cancer dormancy. Additionally, we emphasize the critical role of the tumor microenvironment (TME) in maintaining the dormant state.

## 3 Tumor microenvironment

The TME plays a critical role in various processes, including tumor proliferation, metastasis, angiogenesis, inhibition of apoptosis, immune system suppression, escaping immune surveillance, and tumor dormancy. For distant tumor cells (DTCs) of breast cancer, the establishment of a hypoxic environment in various organs facilitates immune escape, contributing to tumor persistence (15). Prognostic outcomes are closely associated with the patterns of immune cell infiltration in the TME. An unfavorable prognosis is often linked to the infiltration of immune-suppressive cells, such as tumor-associated macrophages (TAMs), T regulatory cells (Tregs), neutrophils, and cancer-associated fibroblasts (CAFs), within the tumor microenvironment (31).

### 3.1 T-cell involvement in breast cancer dormancy and late recurrence

Hypoxia, a hallmark of the TME, plays a pivotal role in inducing cancer cell dormancy while evading immune detection. This process not only prevents T-cell-mediated tumor clearance but also confers resistance to immunotherapy (32). The infiltration of diverse T-cell subsets into the TME has been shown to exert varied effects on the dormancy of breast cancers. Both local and distant breast cancer dormancy are associated with the presence of specific T-cell populations, including CD4+ and CD8+ effector T-cells, as well as effector memory T-cell subsets (33). Among these, a unique subset of CD8+ T-cells, characterized by the expression of CD39, PD-1, and CD8 (CD39+ PD-1+ CD8+), has been identified as a key regulator of breast cancer dormancy. These cells sustain the tumor’s quiescence by secreting pro-inflammatory cytokines, such as TNFα and IFN-γ (34).

CD39, a multifunctional protein, exhibits a complex role in breast malignancies. Functioning as an enzyme, CD39 collaborates with CD73 to catalyze the conversion of adenosine triphosphate (ATP) into adenosine diphosphate (ADP) and cyclic adenosine monophosphate (cAMP), ultimately releasing immunosuppressive adenosine into the TME (35). This enzymatic activity contributes to T-cell exhaustion in tumor-infiltrating CD8+ T-cells, a state characterized by reduced cytotoxicity and immune dysfunction (36, 37). It can be hypothesized that inhibitors targeting CD39 or CD73 may suppress tumor dormancy by preventing the accumulation of extracellular adenosine, thereby restoring the pro-inflammatory functions of immune cells within the tumor microenvironment. Despite the development of numerous T-cell-based immunotherapies, such as checkpoint inhibitors and adoptive cell therapies, these interventions primarily focus on enhancing T-cell cytotoxicity to combat tumor progression. However, their ability to modulate T-cell dynamics and control breast cancer dormancy remains limited. This underscores the need for a deeper understanding of the mechanisms underlying T-cell-mediated tumor dormancy and the identification of novel therapeutic strategies targeting these processes.

### 3.2 Neutrophils in breast cancer dormancy and metastasis

Neutrophils play a significant yet underappreciated role in tumor immunology, contributing to the development, maintenance, generation of polymorphonuclear leukocytes, metastasis, and dormancy of breast cancer (38). As the most abundant immune cells, neutrophils release proteins and DNA-histone complexes, forming neutrophil extracellular traps (NETs), which ultimately lead to neutrophil death (39, 40). In non-specific immunity, NETs act as a defense mechanism by binding to bacteria and inhibiting their migration. However, NETs are also prevalent in the TME, where cancer cells stimulate their release (39, 40). Emerging evidence indicates that NETs significantly contribute to breast cancer metastasis through NF-κB signaling and disseminated tumor cells (DTCs) (41, 42). Furthermore, NETs have been shown to disrupt the dormancy program by activating integrin α3β1 signaling during inflammation (43). In early breast cancers, circulating NETs levels positively correlate with cancer invasiveness and clinical stages, highlighting their role in tumor progression (44, 45). NETs also promote the acquisition of a pro-metastatic phenotype in breast cancer via EMT, upregulating genes associated with pro-inflammatory and pro-metastatic characteristics (46). These findings underscore the critical role of NETs in linking inflammation to cancer dormancy and metastasis. Additionally, NETs may play a significant role in the context of conventional oncology treatments, such as radiotherapy and chemotherapy, which often induce chronic inflammation. For instance, conditions like osteomyelitis and infectious pneumonia, resulting from hypogammaglobulinemia after radiotherapy and chemotherapy, lead to increased NET production in inflamed organs. This creates an environment conducive to the colonization of DTCs, potentially facilitating metastatic spread.

### 3.3 Macrophages in breast cancer dormancy and metastasis

Macrophages are versatile immune cells that influence various aspects of immunity and are essential mediators of tissue homeostasis. Among the cellular components of the TME, tumor-associated macrophages (TAMs) represent a critical population (47). Activated macrophages can adopt two primary phenotypes: M1 (classically activated macrophages) and M2 (alternatively activated macrophages). M1 TAMs are stimulated by type 1 T helper (Th1) cells, which produce cytokines such as IFN-γ, TNF, LPS, and GM-CSF. These M1 TAMs secrete pro-inflammatory interleukins (e.g., IL-1, IL-2, IL-6, IL-12), TNF-α, and chemokines (e.g., CXCL9, CXCL10), thereby promoting antitumor immunity (47). In contrast, M2 TAMs are activated by type 2 T helper (Th2) cells, which secrete cytokines like IL-8, IL-6, and VEGF-A, fostering tumor growth (47).

Recent studies have demonstrated that M1 phenotype TAMs can reverse the dormant state of breast cancer and enhance tumor sensitivity to carboplatin via exosome-mediated mechanisms (48). Conversely, the M2 phenotype is acquired through gap junctional intercellular communication (GJIC) with cancer stem cells (CSCs), resulting in cycling quiescence, reduced proliferation, and carboplatin resistance (48, 49). While M1 TAMs can inhibit tumor progression to a certain extent, excessive TAM infiltration into the TME is associated with tumor microinvasion (50–52). Strategies to suppress tumor growth and dormancy may involve reducing TAM infiltration or promoting the polarization of M2 TAMs toward the M1 phenotype.

Itaconate acid, a novel immune metabolite derived from cis-aconitic acid via the citric acid cycle and catalyzed by Irg1, has been shown to be highly expressed in M2 macrophages, where it exhibits anti-inflammatory properties (53, 54). Interestingly, recent studies have revealed conflicting roles of Itaconate acid in breast cancer. On one hand, Itaconate acid enhances reactive oxygen species (ROS) activity, promoting tumorigenesis and cancer spread (53). On the other hand, Itaconate acid has been shown to inhibit the progression of estrogen receptor-positive (ER+) breast cancers by reprogramming tumor biochemical pathways and inducing abnormal metabolism (55). In estrogen receptor–positive (ER^+^) breast cancer, cellular metabolism is more dependent on oxidative phosphorylation, lipid metabolism, and tightly regulated anabolic pathways. In this context, itaconate-mediated inhibition of succinate dehydrogenase (SDH) and lipid metabolism may critically impair biomass synthesis, ultimately leading to cell death. By contrast, TNBC is typically more glycolytic and metabolically adaptable, enabling partial circumvention of SDH inhibition. TNBC growth is also more reliant on pro-tumorigenic inflammatory macrophage activity, suggesting that macrophage-derived itaconate could paradoxically promote tumor progression by suppressing anti-tumor immune responses. Within the immune microenvironment, the anti-inflammatory effects of itaconate may facilitate immune evasion in highly inflamed, immune-sensitive tumors. However, in metabolically vulnerable ER^+^ tumors, the direct metabolic blockade imposed by itaconate may outweigh the potential immunosuppressive effects, resulting in an overall anti-tumor outcome (53, 55). These findings suggest a complex interplay between Itaconate acid, macrophage polarization, and cancer metabolism, which may offer a novel avenue for therapeutic intervention. However, the precise molecular mechanisms underlying Itaconate acid’s dual effects in cancer remain to be fully elucidated.

### 3.4 Natural killer cells

**Figure 1:**
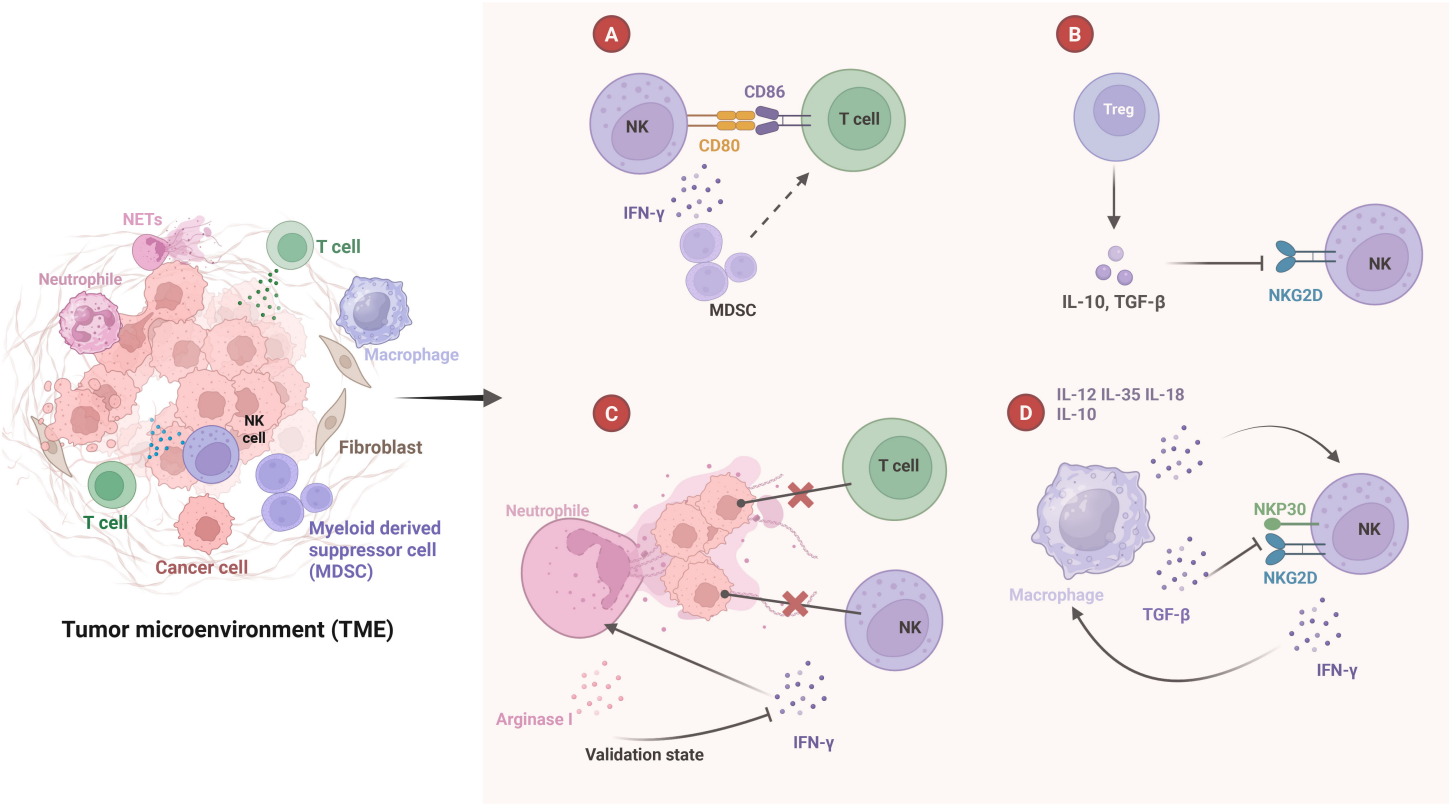
Crosstalk between NK cells and other immune cells in tumor microenvironment. **(A)** NK cells express CD80 and CD86 on T cells to assist in T cell activation and INF-γ secretion. This helps reprogram myeloid-derived suppressor cells (MDSCs) into a mature phenotype, indirectly stimulating T cell activation. **(B)** Treg secrete IL-10 and TGF-β, which inhibit NKG2D expression on the surface of NK cells and inhibit NK cell function. **(C)** NETs prevent T cells and INF-γ from contacting and killing cancer cells, and also promote neutrophil survival. However, neutrophils secrete arginase I, which inhibits NK cells from releasing INF-γ. **(D)** Macrophages secrete various cytokines, including IL-12, IL-35, IL-18, and IL-10. These cytokines promote the proliferation and activation of NK cells. TGF-β inhibits the expression of NKG2D and NKP30 on the surface of NK cells. Additionally, the INF-γ secreted by NK cells stimulates the activation of macrophages.

Natural killer (NK) cells are innate immune cells that play a significant role in the TME alongside other immune components, contributing to cancer metastasis (56, 57). Recent studies have shown that IL-15 induces NK cells to active metastatic dormancy in the liver via INF-γ, while hepatic stellate cells secrete CXCL12 to antagonize NK cell activity (58). This inhibition is analogous to the role of fibroblasts in reducing T-cell infiltration within the TME (18, 59). NK cells also exert a profound impact on immune cells in the TME, exhibiting a unique ability to control and eliminate tumor cells within the immune system (**Figure 1**) (57).

## 4 Gene expression

Recent studies have demonstrated that genetic alterations are essential but not sufficient for the initiation and progression of cancer. Of particular interest, PAQR8 gene expression has been identified as both necessary and sufficient for effective breast cancer recurrence in mice. PAQR8 achieves this by downregulating HER2, a receptor tyrosine kinase positively associated with the activation of Akt, Myc, and WNT1 signaling pathways. These findings suggest that tumor dormancy and recurrence are regulated by epigenetic mechanisms, which confer chemoresistance and reduce cAMP levels in the Gi cycle (60). Furthermore, pro-dormancy programs are orchestrated by NR2F1 and AEPB8, which also contribute to chemoresistance by evading chemotherapy-induced cell death (52, 61). Notably, the overexpression of AEPB1 in breast cancers has been shown to stimulate N-cadherin expression while concomitantly suppressing E-cadherin expression (62). This shift in cadherin expression is indicative of the EMT, a process critical for cancer metastasis and tumor dormancy.

### 4.1 NR2F1

NR2F1, an orphan nuclear receptor belonging to the steroid/thyroid hormone receptor superfamily, forms dimers to bind specific DNA repeats and recruit transcription factors, thereby regulating transcriptional processes. NR2F1 is recognized as a key dormancy promoter, as it drives the expression of specific cancer dormancy markers, such as SOX9 and RARβ, within the TME (63). This receptor is positively regulated by the p38α signaling pathway and negatively controlled by HER2 and WNT4 signaling. Depletion of NR2F1 in early-stage breast cancers leads to reduced expression of EMT regulators, including TWIST1 and ZEB1 (64). Interestingly, NR2F1 expression is re-upregulated in metastatic or resuscitated breast cancer cells, suggesting its dynamic role in cancer progression (64). In HER2^+^ breast cancer, downregulation of NR2F1 correlates with decreased E-cadherin expression and activation of the WNT-dependent β-catenin pathway (65). Notably, NR2F1 expression was more prominently expressed in inflammation-associated CAFs than in the tumor cells. Whether there is a more profound effect between them remains to be studied. This observation underscores the critical role of the tumor microenvironment in shaping cancer dormancy and highlights the epigenetic interplay between tumor cells and their surrounding milieu. Importantly, high expression of NR2F1 is associated with low cell proliferation, making it a key indicator, along with Ki67 (a cell proliferation marker), for diagnosing dormancy in DTCs in clinical practice (66).

### 4.2 ZFP281

ZFP281, a krüppel-like zinc finger transcription factor, serves as a biomarker for early mesenchymal-like (M-like) alterations in breast cancers. It is essential for maintaining cancer dormancy in mice by driving the expression of CD11 (a Type II cadherin) (7, 8). Studies have demonstrated that ZFP281 suppresses osteogenic differentiation and limits growth in mouse embryonic stem cells (ESCs), but it is absent in terminally differentiated human tissues (67). In M-like breast cancer cells, ZFP281 is induced to become activated, predominantly in dormancy-phenotype DTCs rather than in proliferative metastatic cells (67). High expression of ZFP281 is associated with inhibited breast cancer proliferation, suggesting its role in promoting the entry of cancer cells into a dormant state. These findings highlight the dual role of ZFP281 in regulating both cancer dormancy and metastatic potential.

### 4.3 Biglycan

Biglycan (BGN) is a small proteoglycan consisting of a 42kDa core protein with chondroitin sulfate and dermatan sulfate side chains. These side chains are significantly upregulated in dormant breast cancer cells (68). BGN is predominantly expressed by CAFs in the tumor microenvironment and interacts with Toll-like receptors (TLR2 and TLR4), which associate with TGFβ/Snail and TNFα/NF-κB signaling pathways to regulate EMT. This interaction is closely linked to cancer stem cell properties and metastatic potential (69, 70). In MDA-MB-231 breast cancer cells, BGN inhibits cancer cell growth and reduces proliferative metastasis (71). Furthermore, BGN negatively correlates with CD8^+^ T cell infiltration in the TME, thereby promoting cancer dormancy (72). BGN uniquely functions within TME, where it is highly expressed in dormant breast cancers. It exhibits a ligand-like effect by activating downstream signaling pathways, including ERK2 and NF-κB, to regulate tumor cell quiescence (72). While BGN shows promise as a tumor marker, its precise mechanism of action in cancer dormancy remains to be fully elucidated.

## 5 Non-coding RNA

Non-coding RNA (ncRNA) represents a critical component of epigenetic regulation, encompassing various RNA species such as long non-coding RNA (lncRNA), microRNA (miRNA), circular RNA, and other RNA clusters that do not directly encode proteins. ncRNA exerts its regulatory effects on gene expression through mechanisms involving DNA-RNA or RNA-RNA complementation, as well as enzymatic reactions. Furthermore, ncRNA plays a pivotal role in the EMT and MET of cancer cells during cancer dormancy by modulating gene expression, signaling pathways, and metabolic processes. A notable mechanism by which tumor cells adapt immune cells to the TME involves the use of exosomes as carriers for miRNAs, facilitating intercellular communication and miRNA transfer between cells.

### 5.1 MicroRNA

MicroRNAs (miRNAs) are small, non-coding RNAs that regulate gene expression post-transcriptionally by binding to the 3’ untranslated regions (UTRs) of target mRNAs, thereby controlling cell phenotype. miRNAs can interact with target cells via gap junction intercellular communication (GJIC) and exosome-mediated transfer (73). The presence of specific miRNA families in the TME has been identified, and their impact on tumor dormancy has been demonstrated. The role of miRNAs is bidirectional: tumor-associated stromal cells or polymorphonuclear supporting cells can release specific miRNAs, such as miR-21, miR-23b, miR-155, miR-27, miR-197, miR-222, and miR-223, into the TME via exosomes or intercellular communication junctions. These miRNAs influence the expression of genes in cancer cells. Conversely, tumor cells can release miRNAs, such as miR-9, to modulate stromal cells, thereby maintaining a tumor-permissive microenvironment. Certain miRNAs can alter signaling pathways by targeting specific enzymes. For example, the miR-205/31 cluster specifically targets ubiquitin-conjugating enzyme E2 N (UBE2N) to suppress the NF-κB signaling pathway (**Table 1**).

**Table 1:** The roles of miRNA in breast cancer dormancy.

MicroRNA	Objectives and sources	Function	Ref.
miR-9	Target fibroblasts EFEMP1 form TNBC	-inhibit EFEMP1 -acclimate the fibroblasts to the tumor -interfere SOCS3 and promote MDSC -activate JAK/STAT	(74–76)
miR-23b	Target cancer MARCKS from bone marrow-derived MSCs	-promote cancer dormancy and decreased proliferation and invasion	(77)
miR-27/197/222/223	Target cancer CXCL12 gene from bone marrow	-lead to decreased proliferation and arrest cell cycle -promote breast cancer stem cell differentiation and dormancy.	(78)
miR-205/31	Target cancer ubiquitin conjugating enzyme E2 N gene form mesenchymal stromal cells	-inhibit NF-κB -upregulate MMP1 E-cadherin -inhibit cancer cell proliferation and metastasis -make cancer dormancy directly	(79, 80)
miR-155	Target cancer C/EBP-β from exosomes	-down-regulates FOXO expression -make cancer chemically resistant	(81)
miR-200b/200a/429	Mice	-reduce Vim, Snai1, Twist1, Twist2, and Zeb1 expression -inhibit cancer metastasis and induces cancer dormancy	(82)
miRNA-122	Target PMN non-tumor cells form breast cancer	-construction of PMN -promote metastasis and dormancy	(25)

### 5.2 Exosomes

Exosomes, as intraluminal vesicles formed by the fusion of exocytic vesicles with the cell membrane, play a pivotal role in intercellular communication. These nanovesicles contain diverse molecular components, including mRNAs, proteins, and miRNAs, which are derived from the parent cell (83). In our review, we observe that exosomes predominantly carry nucleic acids, which leads us to classify them under the broader category of ncRNAs. Exosomes are increasingly recognized for their involvement in tumor development, particularly in the context of tumor dormancy (10, 84). In cancer, including breast cancers, significant alterations in the Warburg effect and mitochondrial DNA (mtDNA) have been observed, including mutations, deletions, and polymorphisms, which often result in deficiencies in oxidative phosphorylation (OXPHOS) (85–87). CAFs, which produce exosomes containing the complete mitochondrial genome, have been shown to induce tumor quiescence restoration in breast cancer with reduced drug resistance, which is associated with the oxidative phosphorylation of dormant cells being restored (88). Additionally, MSCs have been demonstrated to regulate cancer cell dormancy in bone through exosome-mediated mechanisms (89). BM-MSC-derived exosomes have an increased number of miRNAs, and overexpression of miR-23b in BM2 cells induces a dormant phenotype by inhibiting the target gene MARCKS, which encodes proteins that promote cell cycle and motility (77). Recent evidence highlights that breast cancer can dedifferentiate into CSCs and acquire drug resistance after colonizing the bone marrow, a process mediated by exosomes derived from MSCs (10, 77, 90). Beyond their role in cell communication and material transfer, exosomes have shown promise in drug delivery systems, particularly through exosomes derived from NK cells (91). Furthermore, specific exosomes, such as LINC00657 from breast cancers, have been shown to activate M2 macrophages, thereby promoting tumor progression (92). The therapeutic potential of exosomes is vast but not yet fully elucidated. Exploring the use of autologous or synthetic exosomes as a treatment modality for breast cancer represents an emerging and promising area of research.

### 5.3 Long non-coding RNAs and piwi-interacting RNAs

Recent experimental evidence has demonstrated that certain lncRNAs significantly influence breast cancer progression, particularly in relation to tumor dormancy. Among these, the lncRNA NR2F1-AS1, also known as NAS1, has garnered attention due to its upregulation in breast cancer stem-like cells. This RNA is evolutionarily conserved across species and exhibits ten transcript variants located on chromosome 5q15. NAS1 is known to interact with the NR2F1 protein and recruit the RNA-binding protein PTBP1, thereby facilitating the translation of NR2F1 (93, 94). Additionally, NAS1 functions as a molecular sponge for several miRNAs, including miR-641 and miR-23-3p. The regulation of miR-641, a tumor suppressor, is closely associated with NAS1 levels, exhibiting a negative correlation (93, 94). Furthermore, miR-23-3p has been shown to promote EMT and metastasis in breast cancer by targeting ZEB2 (95). NR2F1 itself plays a critical role in inhibiting the transcription of ρNp63, thereby promoting EMT and cancer dormancy (96). NAS1 has also been associated with the recurrence of ER+ breast cancer, where it appears to activate key regulators such as HIF1α, VEGFA, and ICAM-1, thereby enhancing processes associated with EMT, hypoxia, and inflammation (97).

Moreover, NAS1 has been associated with promoting angiogenesis and metastasis in breast cancer, potentially through its interaction with miR-336-3p and subsequent modulation of the IGF-1R/ERK signaling pathway (98). Given its unique pathophysiological characteristics, NAS1 emerges as a novel biomarker for monitoring tumor progression and a potential therapeutic target for inhibiting cancer dormancy in breast cancer (99).

Piwi-interacting RNAs (piRNAs), a distinct class of non-coding RNAs, play a critical role in the regulation of gene expression. These small RNA molecules, typically 26-32 nucleotides in length, are specifically associated with Piwi family proteins. They are abundant and enriched in mammalian germ cells. While there is currently no direct evidence linking piRNAs to breast cancer cell dormancy, their potential significance in breast cancer research and therapy appears promising. Further investigation into piRNAs may provide critical insights into tumor progression mechanisms and therapeutic strategies.

## 6 Cell adhesion molecule

Cell adhesion molecules (CAMs) are pivotal in maintaining tumor architecture, regulating cancer cell dormancy, and modulating metastatic behavior. They belong to the broader category of human leukocyte differentiation antigens (HLDA, CD antigens) and are classified into four major families: the immunoglobulin superfamily (IgSF)—including CD4, PD-1, PD-L1, and CTLA-4; the integrin family—such as VLA-4 and LFA-1; the selectin family—CD62L, CD62E, CD62P; and the cadherin family, comprising E-cadherin, N-cadherin, P-cadherin, and R-cadherin. Other unclassified adhesion molecules also contribute to the intricate tumor microenvironment. Gap junctions, primarily composed of connexin proteins, have been implicated in both the metastatic dissemination of CTCs and the maintenance of dormancy in CSCs (100, 101). One notable discovery in this area is the role of Kindlin-1, a focal adhesion protein implicated in breast cancer. Knockdown of Kindlin-1 leads to reduced recruitment of tumor-infiltrating regulatory T cells (Tregs) and attenuated immunosuppressive activity, primarily through downregulation of interleukin-6 (IL-6) secretion. This, in turn, facilitates tumor regression (102). During EMT, selective downregulation of cadherins—particularly E-cadherin—is a hallmark, leading to the loss of cell polarity and cell–cell junctions, thereby facilitating metastatic dissemination (98, 99). However, given the essential roles of cadherins in normal tissues, directly targeting them remains a therapeutic challenge. Of particular interest in the context of cancer dormancy and metastasis is N-cadherin, a Type I cadherin with established oncogenic potential.

### 6.1 N-cadherin

N-cadherin (neural cadherin) has emerged as a critical mediator in cancer progression and is frequently associated with mesenchymal phenotypes and TAMs (103, 104). Functionally, it enhances cellular motility, survival, and invasive potential. Aberrant expression of N-cadherin has been linked to diverse oncogenic processes, including cellular transformation, evasion of apoptosis, neovascularization, and metastatic spread, particularly under conditions that support cancer dormancy (105). In breast cancer, N-cadherin plays a central role in mediating gap junction intercellular communication (GJIC) between dormant breast cancer cells and the bone marrow microenvironment. Specifically, N-cadherin is expressed both in the endosteal niche and in hematopoietic stem cells (HSCs). Knockdown of N-cadherin in HSCs leads to increased proliferation *in vitro* and impaired homing and retention in the endosteal region *in vivo* (101, 106).

Furthermore, Notch2^+^ breast cancer cells (e.g., MDA-MB-231) exhibit elevated N-cadherin levels, which facilitates adhesion to spindle-shaped N-cadherin-positive osteoblasts (SNOs). This interaction mimics the HSC niche and contributes to CSC dormancy through activation of the Jagged-1/Notch signaling pathway (107). In this context, breast cancer cells may “educate” or “domesticate” osteoblasts within the niche to preserve a dormant phenotype, indicating a highly dynamic crosstalk between tumor cells and bone-derived stroma (108). Clinically, overexpression of N-cadherin is frequently observed in invasive and metastatic breast tumors and correlates with poor prognosis. Interestingly, elevated levels of N-cadherin mRNA have been detected in the peripheral blood of patients and may serve as a potential liquid biopsy biomarker for early detection of metastasis. A pilot study has suggested that circulating N-cadherin mRNA levels may indicate the emergence of new metastatic lesions, particularly following dormancy escape (109). Compared with circulating tumor cells, N-cadherin can be used as an indirect indicator of metastatic potential, but it provides us with new ideas and research methods. N-cadherin is predominantly expressed in neural and stromal cells, and its upregulation via epithelial–mesenchymal transition (EMT) endows breast cancer cells with enhanced invasive potential. However, there is currently a lack of clinical studies directly comparing circulating tumor cells (CTCs) with N-cadherin mRNA expression in the context of dormancy escape. Moreover, the clinical feasibility of mRNA detection remains substantially lower than that of protein detection. Therefore, future studies should focus on quantifying N-cadherin protein levels—such as through enzyme-linked immunosorbent assay (ELISA)—to evaluate their association with dormancy escape in breast cancer. Such an approach would not only provide prognostic insights but also align more closely with clinical cost-effectiveness and feasibility.

### 6.2 Leukemia inhibitory factor receptor

We summarize the role of a breast cancer suppressor receptor (LIFR, CD118), part of the leukemia inhibitory factor (LIF, IL-6 family), which is inhibited in bone under low oxygen tensions and exhibits distinct influences in other cancers. In breast cancer, overexpression of LIF, predominantly from cancer-associated fibroblasts, is observed. LIF, belonging to the type I cytokine receptor family, is a multifunctional cytokine activated by various cytokines, including ciliary neurotrophic growth factor (CNTF), oncostatin M (OSM), cardiotrophin-1 (CT1), and corticotrophin-like cytokine (CLC). It has been demonstrated that LIFR can promote cellular differentiation, proliferation, and survival in both adult and embryonic tissues. This receptor complex comprises a high-affinity transducer subunit, gp130, which facilitates the transmission of its effects. However, in cancer contexts, parathyroid hormone-related protein (PTHrP) is known to down-regulate LIFR. This down-regulation of LIFR may contribute to the progression of cancer cells out of dormancy, increasing their aggressiveness and proliferation, and thereby promoting tumor growth (110, 111). YAP, a proto-oncogene, is activated by LIFR in the Hippo pathway. LIFR activation inhibits cancer metastasis, while its downregulation activates YAP, promoting migration, invasion, and metastasis (112, 113). Reports indicate that HDAC inhibitors can stimulate LIFR, leading to the acetylation of the LIFR promoter histone, thereby enhancing its expression and causing cancer cell proliferation while preserving dormancy, albeit with more intrusive effects (111, 114). In bone metastases of breast cancer, LIFR knockdown disrupts the dormancy phenotype, with STAT3 being an essential factor in LIFR signaling, and LIF upholds the dormancy phenotype through the LIFR: STAT3: SOCS3 pathway (115). The LIFR: STAT3: SOCS3 axis inhibits WNT signaling through the degradation of β-catenin (116). In ER^+^ breast cancers, nuclear p21-activated kinase 4 (nPAK4) targets LIFR, augmenting the PAK4-ER axis-mediated bone metastases, and functions as a novel repressor of ERα-mediated transactivation, operating in an E2-dependent manner (117). The nuclear PAK4-Erα-LIFR axis facilitates bone metastasis of ER^+^ breast cancers by initiating an EMT program, where high expression of nPAK4 is often associated with a poorer prognosis (118). This suggests that nPAK4 may modulate the dormancy or CSC-like phenotype in breast cancers through LIFR, representing a potential target or novel tumor marker in cancer therapy.

## 7 Signaling pathway of WNT

**Figure 2:**
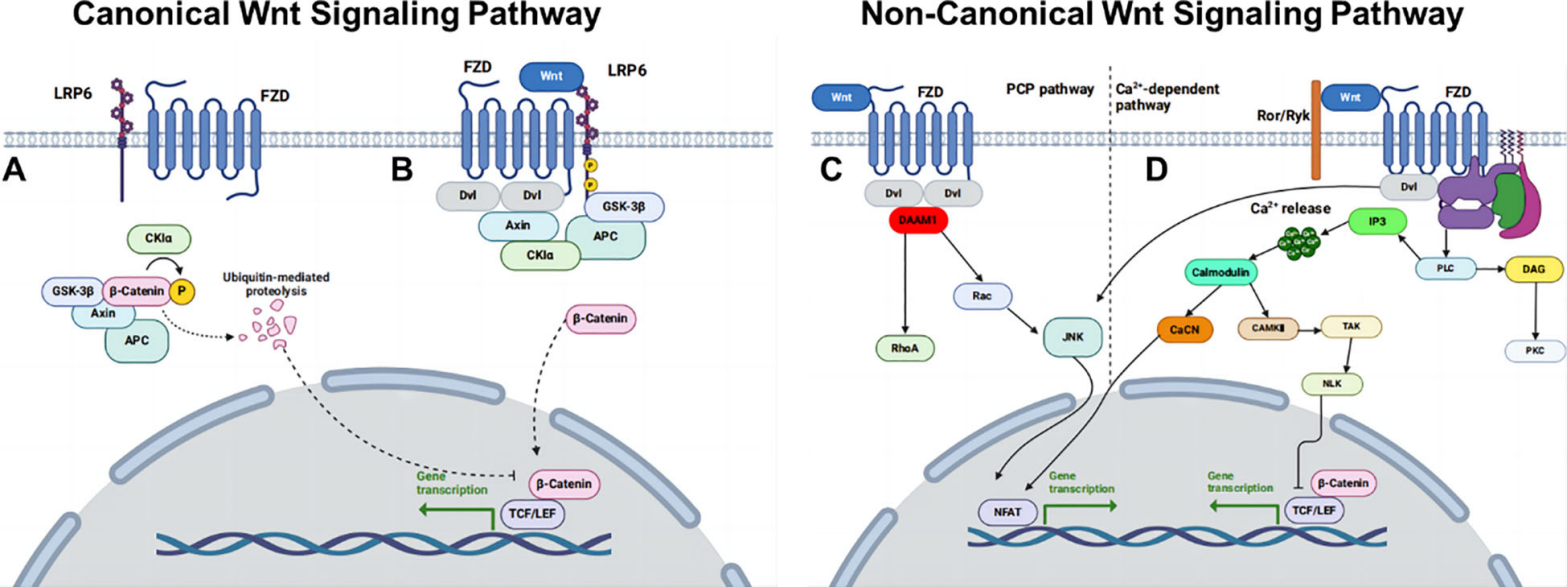
Overview of WNT signaling pathways. **(A)** Canonical WNT pathway off. **(B)** Canonical WNT pathway on. **(C)** Non-canonical WNT/PCP pathway and **(D)** non-canonical WNT/Ca^2+^ pathway.

Wnt signaling is a key regulator of developmental and postnatal processes (**Figure 2**) (119). Reviewing the relationship between partial dormancy factors and WNT signaling, PAQR8 expression has been shown to reduce WNT1 activity in HER2^+^ breast cancer models, while the loss of NR2F1 activates WNT signaling and subsequently inhibits E-cadherin expression. Activation of the LIFR: STAT3: SOCS3 pathway also results in the inhibition of WNT activity. This analysis reveals a significant relationship between the WNT signaling pathway and breast cancer dormancy, mediated by effector molecules regulating the expression of cell cycle and cell polarity-related proteins, which in turn influence cell differentiation, localization, and polarity (119).

Embryonic stem cells rely on WNT proteins to prevent ectodermal differentiation and maintain totipotent differentiation (120). CSCs are dormant cancer stem cells with low replication rates and can differentiate into high-grade tumor cells. Up-regulation of NOTCH and WNT signaling genes following ectopic COX-2 expression or treating COX-2 low T47D and MCF-7 cells with the nonselective EP ligand PGE-2 or a selective EP4 agonist PGE1OH. This resulted in the down-regulation of mRNA of E-cadherin and up-regulation of mesenchymal markers’ mRNA, such as N-cadherin, TWIST1, and SNAIL (121).

The AMPK activator metformin suppresses breast cancer cell growth by downregulating disheveled segment polarity protein 3 (DVL3) and β-catenin expression levels. Dishevelled (DVL), a key activator of the WNT/β-catenin signaling pathway, is overexpressed in various tumors (122). AMPK modulates WNT signaling through multiple mechanisms. Its upstream kinase, liver kinase B1 (LKB1), when inactivated, reduces AMPK activity and thereby promotes tumor progression. Conversely, activated AMPK phosphorylates the deubiquitinase USP10, enhancing the deubiquitination and stabilization of the scaffold protein Axin1. This phosphorylation also strengthens the interaction between USP10 and β-catenin, facilitating the phase transition of β-catenin. Both mechanisms act in parallel to attenuate WNT/β-catenin signaling amplitude, thereby suppressing tumor growth, as demonstrated in clinically relevant colorectal cancer models (123). The regulatory interplay between USP10 and the AMPK/WNT signaling axis has been extensively characterized in colorectal cancer; however, its relevance in breast cancer, particularly in the context of tumor dormancy, remains largely unexplored. Elucidating this mechanism in breast cancer could represent a promising avenue for future research.

In the normal mammary gland, Notch and WNT signaling exhibit a synergistic relationship, mediating intricate interactions between mammary stem cells (MaSCs) and the macrophage-rich stromal niche. Specifically, the Notch ligand Delta-like 1 (Dll1), expressed on MaSCs, engages macrophages in the niche, prompting them to secrete WNT ligands (WNT10A, WNT16, and WNT3). These WNT signals are critical for sustaining MaSC numbers and functional activity. Although this Notch–WNT axis has been characterized in normal murine mammary development, its potential role in breast cancer—particularly in regulating cancer stem cell behavior and tumor dormancy—warrants further investigation (124).

## 8 Metabolism of dormancy

Recent studies have demonstrated a significant association between obesity and the metastatic potential of postmenopausal breast cancers (125, 126). These factors collectively establish a favorable microenvironment for both local tumor progression and systemic dissemination. Notably, endocrine dysregulation, often resulting from hormone therapy resistance or other endocrine pathologies, can impair fat metabolism and increase body weight, thereby enhancing the recurrence of dormant breast cancers (127).

For patients with early-stage breast cancers who respond to initial treatment, strict dietary control and monitoring of endocrine status are crucial to maintaining tumor dormancy. Metformin, a sulfonylurea, has shown promise in preserving the dormancy of ER^+^ breast cancer cells by activating the AMPK pathway, which promotes cellular energy metabolism. Additionally, metformin reduces blood sugar levels, potentially inhibiting cancer cell proliferation and enhancing treatment efficacy (128, 129). These findings underscore the importance of metabolic regulation in modulating breast cancer dormancy and recurrence.

In dormant tumor cells, lipid metabolism has been found to be enhanced, and its activity appears to correlate closely with therapeutic outcomes. One key enzyme, acyl-coenzyme A synthetase long-chain family member 3 (ACSL3), facilitates the activation and incorporation of monounsaturated fatty acids into the cell membrane, and its expression is upregulated in disseminated tumor cells (DTCs) (17). Pharmacological inhibition of ACSL3 leads to lipid peroxidation and ferroptosis— a form of regulated, non-apoptotic cell death. These findings suggest that monitoring lipid metabolic pathways may serve as a potential indicator of the quiescent state of tumor cells. In studies involving 5-aminolevulinic acid-based photodynamic therapy (5-ALA-PDT), inhibition of acyl-CoA synthetases (ACSs) has been shown to cause protoporphyrin IX accumulation, thereby reducing treatment sensitivity (16). While lipid metabolism presents a promising avenue for understanding and targeting tumor dormancy, further research is necessary to fully elucidate its clinical relevance.

## 9 Conclusion and future perspective

Tumor dormancy serves as a significant obstacle to breast cancer treatment, with our findings indicating that it plays a pivotal role in the recurrence of advanced breast cancer. The concept of tumor dormancy suggests that the cessation of cancer cell proliferation may contribute to the establishment of cell dormancy, surpassing the conventional therapeutic endpoint. In the context of palliative care for tumor patients, the achievement of dormancy—a state that effectively inhibits proliferation and metastasis—represents an optimal therapeutic goal. Guided treatment represents the highest priority in the study of breast cancer dormancy. On one hand, suppressing dormancy in early-stage cancers may enable precise eradication of cancer cells. Conversely, maintaining cancer cell dormancy may act as a protective mechanism against late-stage recurrence. This dual approach may enhance the management and treatment of breast cancer across various stages.

Dormancy, while often observed, raises several unanswered questions regarding its mechanisms and variability. The study of dormancy is inherently complex, involving multiple temporal and spatial scales—from genetic regulation to the TME. While dormancy is commonly associated with stem cell-like properties, such as low replication and metastatic potential, the exact molecular mechanisms underlying its regulation remain incompletely understood. Recent advancements in *in vitro* studies have revealed the utility of 3D culture systems in simulating tumor cell proliferation and dormancy within the tumor microenvironment (130–132). However, challenges persist in optimizing culture conditions, particularly due to material synthesis complexity, high costs, and restrictive requirements.

The interplay between autophagy and cancer dormancy represents another critical area of exploration. Recent studies suggest that autophagy may serve dual roles in maintaining dormancy. In early-stage cancer, autophagy may act as a defense mechanism, whereas in later stages, it could promote tumor progression (84, 133). Inhibiting autophagy has been shown to potentially induce tumor dormancy by destabilizing the local anoxic environment, which may contribute to the maintenance of cancer cell dormancy. Furthermore, the role of tumor suppressor genes, such as DIRAS3, in initiating autophagy appears crucial. Upon malignant transformation, DIRAS3 reactivation may facilitate autophagy, subsequently inducing tumor dormancy (130, 133–136).

The implications of lncRNAs, circRNA and miRNAs in regulating tumor dormancy are multifaceted (137). Abnormal WNT signaling, which regulates cell cycle progression and phenotypic differentiation, is frequently observed in dormant cells. Similarly, the dysregulation of NF-κB in the TME may contribute to tumor progression. The significance of various components within the TME, including proteoglycans, exosomes, and immune cells like MDSCs and Tregs, remains poorly understood. These elements collectively contribute to the persistence of tumor mass dormancy and immune tolerance associated with dormancy. Additionally, the roles of M2-type macrophages and neutrophils in the TME, particularly through mechanisms such as neutrophil extracellular traps, may influence tumor progression and treatment outcomes.

From the perspective of immunotherapy, CAR-T cell therapy and other chimeric antigen receptor therapies have demonstrated promising potential in treating various cancers, including breast cancer. However, their efficacy against solid tumors remains limited, particularly in the context of dormant breast cancer. This limitation arises from the fact that dormant cancer cells often reside in immune-privileged niches and exhibit downregulated expression of surface receptors, thereby diminishing the effectiveness of receptor-targeted therapies. Tumor vaccines represent another promising approach, as they have shown potential in inducing tumor dormancy by targeting specific elements of the TME, such as MDSCs. Computational simulations have suggested that these vaccines may achieve their intended effects by modulating the TME, though the mechanisms remain complex (138). Recent computational studies have also highlighted the importance of considering non-specific immune cells, which are often co-opted by tumors to maintain the TME. In the TME, recent studies have revealed the significant role of resident microorganisms in TNBC. Notably, *Sphingobacterium multivorum* (*S. multivorum*) has been shown to promote the secretion of CCL20 and CXCL8 by tumor cells, which leads to an increase in regulatory T cells (Tregs) and a decrease in CD8^+^ T cells, thereby facilitating immune evasion. Interestingly, *S. multivorum*, through its metabolite propionyl carnitine, has also been found to inhibit tumor cell growth. These findings highlight the dual role of microenvironment-colonizing bacteria in modulating both immune responses and tumor cell behavior, offering new insights into the regulation of tumor cell quiescence and potential therapeutic strategies (139). Therefore, a more comprehensive understanding of dormancy requires a multidisciplinary approach that integrates insights from molecular biology, oncology, and immunology.

Future research in tumor dormancy must address several key challenges. First, the development of precise, non-invasive biomarkers (e.g., CTCs, NR2F1+ DTCs, exosome miR122) for tumor dormancy remains critical. Such biomarkers could guide clinical decision-making and inform therapeutic strategies. Second, the identification of novel therapeutic targets and intervention points within the TME represents a promising direction. This may involve the exploration of novel molecular mechanisms or the identification of key regulatory nodes that can be targeted to disrupt dormancy. Third, the integration of multidisciplinary approaches, including computational modeling and *in vivo* imaging, 3D dormancy models, and single-cell DTC sequencing, could enhance our understanding of dormancy and its implications for treatment. Finally, the translation of preclinical findings into clinical practice must be supported by robust, randomized controlled trials. As our understanding of tumor dormancy continues to evolve, it is essential to prioritize translational research and clinical validation to maximize the potential impact of new therapies.

In conclusion, the study of tumor dormancy presents significant challenges but also offers profound insights into the mechanisms underlying breast cancer recurrence and metastasis. Continued research is required to unravel the complex interplay between genetic, molecular, and environmental factors that contribute to dormancy. By addressing the gaps in our current understanding and advancing novel therapeutic strategies, we may ultimately pave the way for more effective treatments for breast cancer.
